# P-557. A Cost-Effectiveness Analysis of Chikungunya Virus Vaccination in Brazilian Adults

**DOI:** 10.1093/ofid/ofaf695.772

**Published:** 2026-01-11

**Authors:** Christina Guo, Christopher Xiao, Juliana Paiva, Yin Hong Lo, Emmanuel Drabo

**Affiliations:** Johns Hopkins Bloomberg School of Public Health, Beaumaris, Victoria, Australia; Johns Hopkins Bloomberg School of Public Health, Beaumaris, Victoria, Australia; Johns Hopkins Bloomberg School of Public Health, Beaumaris, Victoria, Australia; Johns Hopkins Bloomberg School of Public Health, Beaumaris, Victoria, Australia; Johns Hopkins Bloomberg School of Public Health, Beaumaris, Victoria, Australia

## Abstract

**Background:**

Chikungunya virus (CHIKV) causes substantial health and economic burden in Brazil. From 2011 to 2020, Brazil reported 3.2 million cases and a $9.8 billion economic burden, experiencing annual epidemics since 2016. Two vaccines exist, but their cost and benefit tradeoffs are unclear. This study evaluates the cost-effectiveness of the IXCHIQ and Vimkunya vaccines for prevention of CHIKV infection in Brazilian adults.

**Figure:**
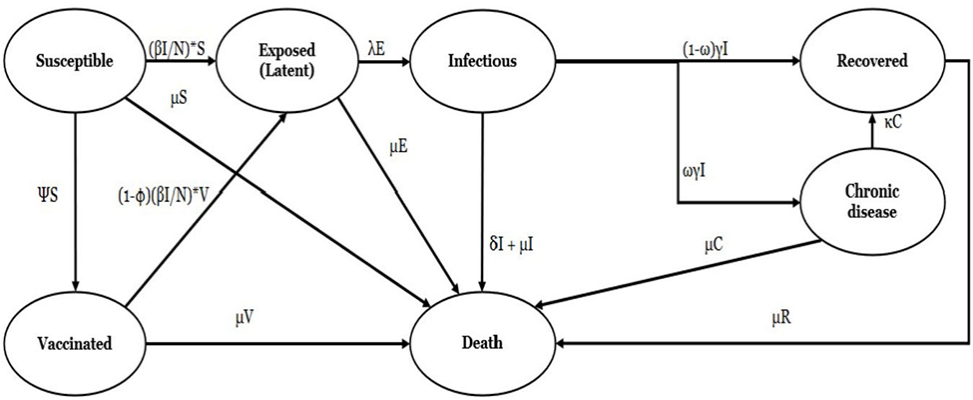

**Figure:**
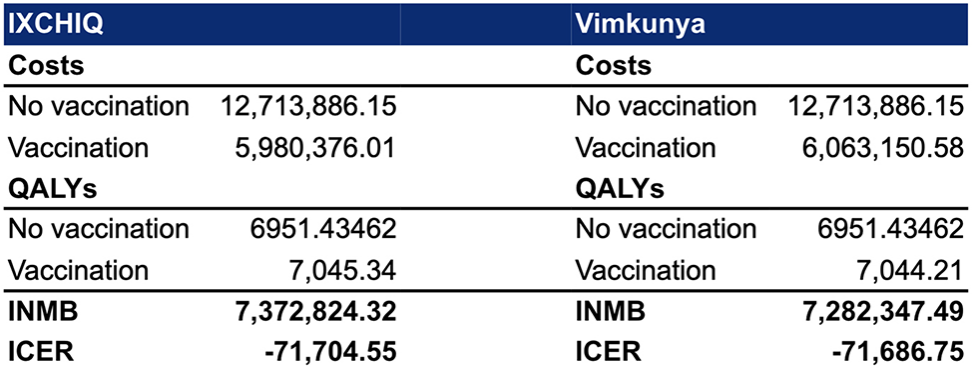

**Methods:**

A SVEIRD model was used to assess the cost-effectiveness of either IXCHIQ or Vimkunya vaccination in a hypothetical cohort of 1000 Brazilian adults over a 10-year time horizon. Costs and quality-adjusted life-years (QALYs) were assessed from a societal perspective. Inputs were derived from published estimates, trial data, and assumptions where data was unavailable. Costs were inflated to 2025 US dollars and both costs and utilities were discounted at 3% annually. The incremental net monetary benefit (INMB) of both vaccines was compared to no vaccination. Parameter uncertainties were assessed with deterministic and probabilistic sensitivity analyses.

**Figure:**
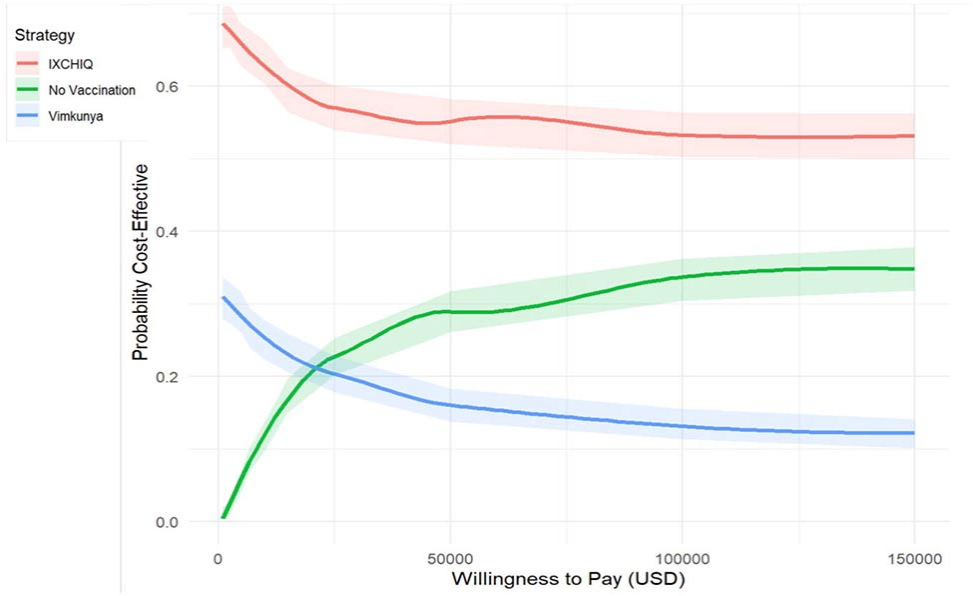

**Figure:**
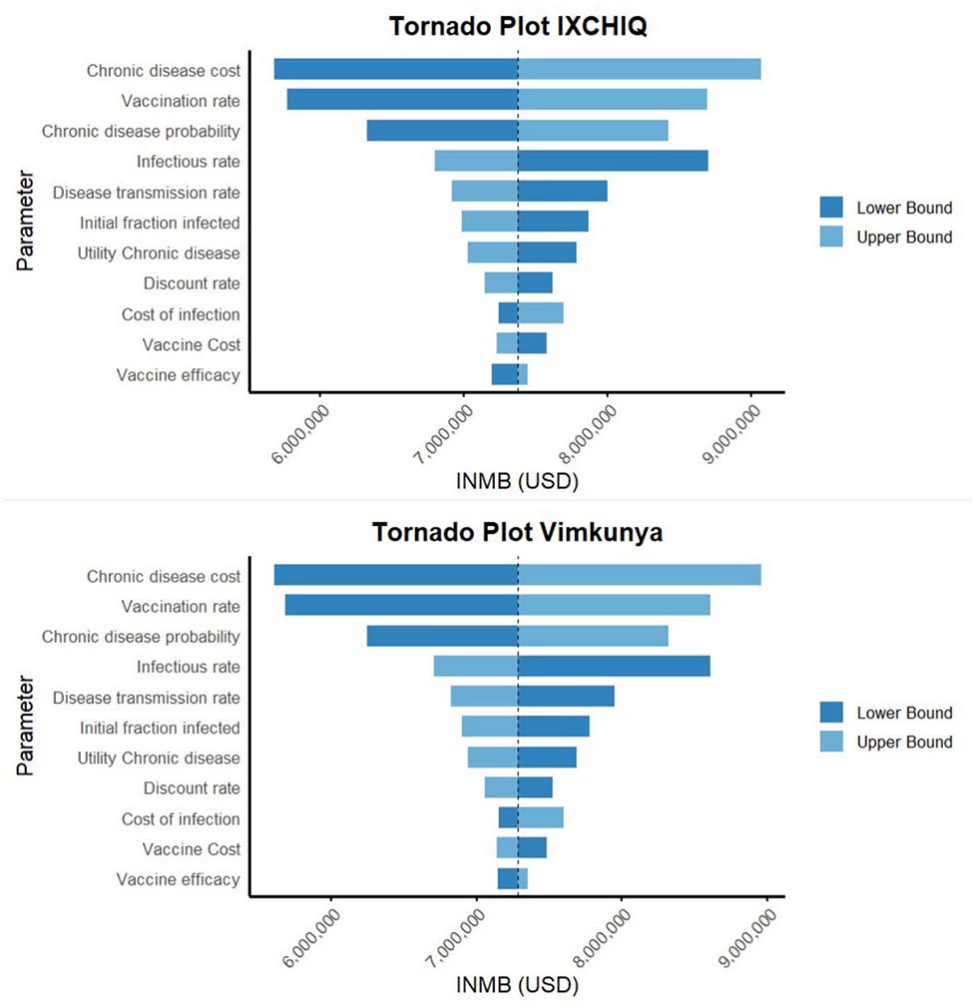

**Results:**

In the base case analysis, IXCHIQ vaccination dominated both Vimkunya and no vaccination. IXCHIQ was more effective (7045.34 QALYs) and less costly ($5,980,376) than Vimkunya (7,044.21 QALYs, $6,063,151) or no vaccination (6,951.43 QALYs, $12,713,886). The INMB was $7,372,824 for IXCHIQ and $7,282,347 for Vimkunya at a willingness-to-pay (WTP) threshold of R$40,000 (Brazilian real) (approximately $6808). Probabilistic sensitivity analysis indicated IXCHIQ is preferred in 64.8% of simulations at a WTP of R$40,000.

**Conclusion:**

Chikungunya vaccination is a cost-effective approach for reducing CHIKV-associated economic and health burden in Brazil, with IXCHIQ slightly preferred over Vimkunya. Although our findings highlight the economic and public health benefits of chikungunya vaccination, better evidence on health-related quality of life and vaccine costs are needed for more robust policy recommendations.

**Disclosures:**

All Authors: No reported disclosures

